# NPC1 promotes the progression of hepatocellular carcinoma by mediating the accumulation of neutrophils into the tumor microenvironment

**DOI:** 10.1002/2211-5463.13951

**Published:** 2024-12-20

**Authors:** Songhai Yang, Jiangming Chen, Kun Xie, Fubao Liu

**Affiliations:** ^1^ Department of Hepatobiliary and Pancreatic Surgery The First Affiliated Hospital of Anhui Medical University Hefei China; ^2^ Department of General Surgery The First People's Hospital of Hefei China

**Keywords:** hepatocellular carcinoma, neutrophils, NPC1, the tumor microenvironment

## Abstract

Hepatocellular carcinoma remains a significant threat to human health. Recent studies have found that the intake of cellular cholesterol contributes to the development and progression of hepatocellular carcinoma, although the exact mechanisms remain unclear. Our analysis of transcriptomic and proteomic databases has identified increased mRNA and protein expression levels of NPC1, a cholesterol intracellular transporter protein, in hepatocellular carcinoma tissues. This increase is significantly associated with a worse prognosis for patients. To corroborate these findings, we performed immunohistochemical staining of NPC1 on liver tissue samples from patients, revealing significantly higher expression levels of NPC1 in hepatocellular carcinoma tissues compared to normal tissues. Subsequent investigations have revealed that NPC1 expression does not significantly influence the proliferation of hepatocellular carcinoma cells *in vitro*. However, it has a substantial inhibitory effect on the progression of hepatocellular carcinoma tumors when observed *in vivo*. Utilizing flow cytometry to monitor cellular changes within the tumor microenvironment has led us to discover that NPC1 plays a crucial role in the regulation of neutrophil recruitment within the tumor. Using further neutrophil depletion experiments, we determined that the role of NPC1 in advancing hepatocellular carcinoma progression truly relies on neutrophils. These observations are further reinforced by a comprehensive analysis of clinical databases alongside immunohistochemistry findings. In conclusion, our research suggests that NPC1's overexpression could contribute to hepatocellular carcinoma progression by promoting neutrophil recruitment, positioning NPC1 as a promising new biomarker and therapeutic target for hepatocellular carcinoma.

## Graphical abstract

Compared to normal tissues, NPC1 expression is significantly elevated in hepatocellular carcinoma tissues. Our findings indicate that NPC1 plays a key role in regulating neutrophil recruitment within the tumor, which is a critical factor in the progression of hepatocellular carcinoma and is closely linked to poor patient outcomes. Overall, our results suggest that NPC1 promotes tumor progression by facilitating neutrophil accumulation.

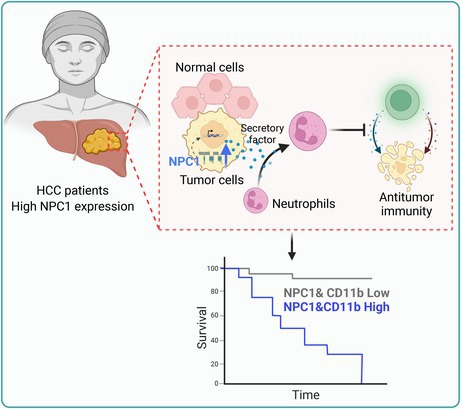

**Abbreviations**
CNHPP: Chinese Human Proteome Project
IHC: histology and immunohistochemistry
LIHC: liver hepatocellular carcinoma
NPC1: Niemann–Pick disease type C1
TCGA: The Cancer Genome Atlas

Hepatocellular carcinoma ranks as the third most prevalent malignant tumor in terms of mortality, following stomach and esophageal cancers. The early symptoms of hepatocellular carcinoma are typically indistinct, resulting in diagnoses frequently occurring at the disease's late stage, thereby bypassing the ideal timeframe for treatment [1, 2]. While there are various treatment options available, such as surgical removal, chemotherapy, and radiotherapy, their effectiveness is limited, particularly in late‐stage hepatocellular carcinoma patients [3]. Surgical removal is an option only for a select few early‐stage patients, and the outcomes of chemotherapy and radiotherapy are not always satisfactory. Even through with advancements in medical technology, such as targeted therapy and immunotherapy, there has been a slight improvement in the survival rates of hepatocellular carcinoma patients [4]. Therefore, it is of utmost importance to identify new biomarkers and therapeutic targets for enhancing our ability to detect and treat hepatocellular carcinoma more effectively.

The development and progression of hepatocellular carcinoma (hepatocellular carcinoma) are linked to various factors. External factors mainly include: hepatitis B virus (HBV) is one of the leading causes of hepatocellular carcinoma, particularly in Asia and Africa [5]; hepatitis C virus (HCV) is also closely associated with the incidence of hepatocellular carcinoma, especially in Western countries [6]. Additionally, prolonged excessive alcohol consumption can lead to cirrhosis, thereby increasing the risk of hepatocellular carcinoma. Individuals with a family history of hepatocellular carcinoma may face higher risks [6]. On the cellular level, anomalies in cholesterol metabolism are a major factor. Cholesterol is a crucial component of cell membranes and is vital for maintaining their integrity and function [7]. Abnormal cholesterol metabolism may promote the growth and proliferation of tumor cells, which often require more cholesterol to support their rapid division.

The NPC1 (Niemann–Pick disease type C1) gene encodes a protein that plays a vital role in cholesterol transport [8, 9]. The NPC1 protein, located on the membranes of lysosomes and endosomes within cells, is responsible for transporting unesterified (free) cholesterol from these organelles [9, 10]. This process is essential for maintaining the balance of cholesterol levels within cells. In the absence of NPC1/2, cholesterol accumulates within the late endosome/lysosome (LE/Lys) lumen, resulting in a cellular cholesterol imbalance. This disruption derails membrane trafficking and impairs communication between organelles, ultimately leading to cellular dysfunction [11, 12].

Several studies have linked distinct NPC1 expression patterns with various types of cancer. Elevated levels of NPC1 have been associated with decreased overall survival in glioma [13], esophageal cancer [14], and ER‐negative breast cancer cells [15]. In esophageal adenocarcinoma, a fusion transcript of NPC1 with a mitotic kinase, maternal embryonic leucine zipper kinase (MELK), was identified in two biopsies [16]. Regarding liver‐related issues, cholesterol and other lipid accumulation can contribute to liver injury during chronic inflammation. Patients with Niemann–Pick disease may be at a higher risk for fibrosis, cirrhosis, and ultimately hepatocellular carcinoma (HCC) development [17]. Additionally, high NPC1 levels in imatinib‐ and daunorubicin‐resistant leukemic cells may undermine the efficacy of anticancer therapies [18, 19]. Inhibition or knockdown of NPC1 has been shown to reduce proliferation, spreading, and migration in several common cancer cell lines [11]. U18666A can directly bind to a site that is within a section of the NPC1 protein called the sterol‐sensing domain. The binding of U18666A to this site blocks the movement of cholesterol out of lysosomes [20]. Given the active role of cholesterol metabolism in the liver, abnormalities in NPC1 function may impact the normal functioning of liver cells, thereby increasing the risk of hepatocellular carcinoma. However, the exact role and mechanisms by which NPC1 might regulate the onset and progression of hepatocellular carcinoma are still unclear.

In our study, utilizing the TCGA database analysis, we found that the mRNA of the intracellular cholesterol transporter NPC1 is highly expressed in hepatocellular carcinoma tissues and is significantly associated with poor prognosis in patients. Further, using proteomics data from hepatocellular carcinoma patient tissues, we observed a significant upregulation in the protein levels of NPC1 in hepatocellular carcinoma tissues, also significantly associated with poor patient outcomes. To validate these findings, we collected tissues from 50 patients and conducted NPC1 immunohistochemical staining. The results similarly indicated that compared to normal tissues, NPC1 expression levels are significantly higher in hepatocellular carcinoma tissues. Subsequent investigations have revealed that NPC1 expression does not influence the proliferation of hepatocellular carcinoma cell lines *in vitro*. However, it has a substantial inhibitory effect on the progression of hepatocellular carcinoma tumors when observed *in vivo*. The results imply that NPC1 could facilitate the advancement of hepatocellular carcinoma via the tumor microenvironment. Utilizing flow cytometry to monitor cellular changes within the tumor microenvironment has led us to discover that NPC1 plays a crucial role in the regulation of neutrophil recruitment within the tumor. This suggests that the modulation of neutrophil recruitment by NPC1 could be a significant factor in the progression of hepatocellular carcinoma. Through additional neutrophil depletion experiments, it was found that the functionality of NPC1 truly relies on neutrophils. And these observations are also reinforced by comprehensive analysis of clinical databases alongside immunohistochemistry findings. In conclusion, our results demonstrate that NPC1 promotes hepatocellular carcinoma progression by mediating the accumulation of neutrophils in the tumor. Our research has provided new potential targets for the diagnosis and treatment target of hepatocellular carcinoma.

## Materials and methods

### Cell line

The Hepa1‐6 cell line was procured from the American Type Culture Collection (ATCC, Manassas, VA, USA). These cells were meticulously cultured in high‐glucose DMEM (Invitrogen/Thermo Fisher Scientific, MA, USA), enriched with 10% fetal bovine serum (Gibco, Grand Island, Waltham, NY, USA), and 1% penicillin–streptomycin (Beyotime Biotechnology, Jiangsu, China), all maintained at a consistent temperature of 37 °C in an environment containing 5% CO_2_.

### Stable cell line constructed

A stable cell line was constructed by first infecting the target cells with lentiviral particles. Lentiviral particles were produced in HEK293T cells, with the virus being collected 48 h following transfection and filtered using 0.45 μm nonpyrogenic filters (Merck Millipore, Billerica, MA, USA). To infect the cells, a mixture of diluted lentivirus and 8 μg·mL^−1^ polybrene (Sigma‐Aldrich, St. Louis, MO, USA) was added to the tumor cells. After 48 h postinfection, puromycin (1 μg·mL^−1^) was added to the culture to select for cells that had successfully integrated the lentiviral vector.

We constructed shRNAs targeting NPC1 using the pLKO.1‐TRC cloning vector (Addgene Plasmid #10878, Cambridge, MA, USA). The cloning procedure is thoroughly described in Protocol Version 1.0 (December 2006), which can be accessed here (https://www.addgene.org/protocols/plko/). The sequences used in this study are as follows: sh1: GCCAAACGATTCGTATGTGAT (corresponding to nucleotides 2598–2618 in NPC1 mRNA, transcript ID: NM_008720.2); sh2: CCCGTCTTACTCAGTTACATA (corresponding to nucleotides 3730–3750 in NPC1 mRNA, transcript ID: NM_008720.2).

### Filipin staining

Hepa1‐6 cells were first fixed using paraformaldehyde to preserve cellular structure and integrity, then permeabilized with a detergent to ensure effective penetration of the staining agent. The cells were subsequently stained with filipin (Sigma), a fluorescent polyene antibiotic that selectively binds to unesterified cholesterol, allowing for the visualization of sterol distribution within the cell membrane and cytoplasm [21]. The staining procedure was carried out according to the protocol outlined by Mukherjee *et al*. [22], which has been widely used in studies to assess cholesterol content and trafficking in various cell types under different experimental conditions. After staining, the hepatocytes were imaged using fluorescence microscopy, and the resulting fluorescence indicated the cellular localization and accumulation of cholesterol. Furthermore, the intensity of filipin staining was quantified by randomly selecting five different fields of view using imagej [Deltavision microscope (GE Healthcare), Marlborough, MA, USA].

### *In vitro* proliferation assay

Hepa1‐6 cells were plated on 96 well plates at 1 × 10^3^ cells per well and cultured at 37 °C under a 5% CO_2_ atmosphere. Subsequently, the cells were subjected to a continuous 5‐day counting analysis, followed by the plotting of the tumor cell growth curve.

### Hepa1‐6 tumor models

Hepa1‐6 cells, amounting to 5 × 10^6^, were carefully subcutaneously implanted into the right flank of male C57BL/6 mice, aged 8–10 weeks (procured from SLAC Laboratory Animal Co.). All mouse experiments were approved by the Ethics Committee of The First Affiliated Hospital of Anhui Medical University (LLSC2021‐12‐30). To monitor tumor growth, measurements were taken of two perpendicular diameters of the tumors in a blinded manner. The size of the tumors was calculated using the formula: 4p/3 × (width/2)^2^ × (length/2). Three weeks subsequent to the tumor inoculation, the tumors were harvested for analysis via flow cytometry. Mice were euthanized using methods of carbon dioxide euthanasia. *In vivo* neutrophil depletion was performed as previously [23]. Briefly, the anti‐Ly6G antibody (1A8, Bio X Cell, West Lebanon, NH, USA) or IgG2a Isotype control (2A3, Bio X Cell) at a dose of 12.5 μg per 100 μL PBS was administered daily through intraperitoneal injection, starting 7 days before Hepa1‐6 cells injection.

### TCGA data analysis

The analysis of TCGA‐related data was conducted with a focus on the expression level of NPC1 in hepatocellular carcinoma tissues. This was primarily achieved using the online platform UALCAN [24], which can be accessed at https://ualcan.path.uab.edu/. To explore the relationship between NPC1 expression and patient survival, survival curves were generated using the Kaplan–Meier Plotter [25], available online at https://kmplot.com/analysis/index.php?p=background. Additionally, the mutation status of NPC1 in hepatocellular carcinoma was investigated using data from Cbioportal, accessible at https://www.cbioportal.org/.

### Chinese human proteome project (CNHPP) data analysis

The analysis of CNHPP‐related data, particularly focusing on liver data, was conducted following the download from the CNHPP Liver Data Portal [5], accessible at http://liver.cnhpp.ncpsb.org/. The primary tools used for this analysis were the R programming language and graphpad. These tools facilitated the examination of NPC1 expression and the generation of survival curves to assess the relationship between NPC1 expression and patient survival.

### Histology and immunohistochemistry (IHC)

Fifty human hepatocellular carcinoma tissue samples were obtained from The First Affiliated Hospital of Anhui Medical University. Prior to sample collection, written informed consent was obtained from all participants. This study was conducted with the approval of the Ethics Committee of The First Affiliated Hospital of Anhui Medical University, under the reference number P2021‐12‐30. Furthermore, all procedures involving human materials or data adhered to the principles outlined in the Declaration of Helsinki.

Immunohistochemistry was performed as described in previous studies [26]. The sections were treated with 3,3′‐Diaminobenzidine Tetrahydrochloride (DAB) (DA1010, Solarbio Life Science, Beijing, China) and then counterstained with hematoxylin (H8070, Solarbio Life Science). Whole‐slide images were captured using the PANNORAMIC 1000 (3DHISTECH Ltd., Budapest, Hungary). Quantitative analysis was conducted for NPC1 (13926‐1‐AP, Proteintech, Rosemont, IL, USA) and CD11b (21851‐1‐AP, Proteintech), all at specified dilution ratios. The intensity of the IHC chromogen was measured as previously outlined [26]. A pathologist evaluated the IHC scores of the stained proteins, and the percentage of positive cells in the tissues was used to determine the IHC score, with the scoring system defined as follows: 0 for 0–5%, 1 for 5–25%, 2 for 25–50%, 3 for 50–75%, and 4 for more than 75% positive cells.

### Tumor‐infiltrating immune cell analysis

We utilized timer version 2.0 to conduct a thorough analysis of immune infiltrates in hepatocellular carcinoma using timer version 2.0 [27], an accessible online resource found at http://timer.cistrome.org/. Before proceeding to investigate tumor‐infiltrating Neutrophil cells among TCGA‐LIHC patients, we meticulously selected all parameters in strict adherence to the instructions detailed within the tool's manual.

### Statistical analysis

graphpad prism version 8.0 (GraphPad Software Inc, La Jolla, CA, USA) served as the tool for conducting statistical analyses of our findings. The significance of gene expression variations was assessed employing two‐way ANOVA. Furthermore, survival outcomes were analyzed using the Log‐rank test, whereas additional analyses were performed with two‐tailed *t*‐tests. We adopted a *P*‐value threshold of less than 0.05 to denote statistical significance.

## Results

### NPC1 mRNA is highly expressed in hepatocellular carcinoma and is associated with the progression of the disease and poor prognosis in patients

It is widely recognized that disruptions in cholesterol homeostasis are associated with lower overall survival rates and an increased risk of poor prognosis following first‐line surgical treatment. In this study, we have highlighted the key proteins involved in cholesterol regulation, including LDLR, NPC1, SCAP, SREBF2, HMGCR, CYP7A1, among others (Fig. S1A). Drawing on previously published data [23], we observed that NPC1 is significantly upregulated in hepatocellular carcinoma (HCC) tumor tissue compared to nontumor tissue (Fig. S1A). Thus, we chose NPC1 as the primary focus of our investigation.

**Fig. 1:**
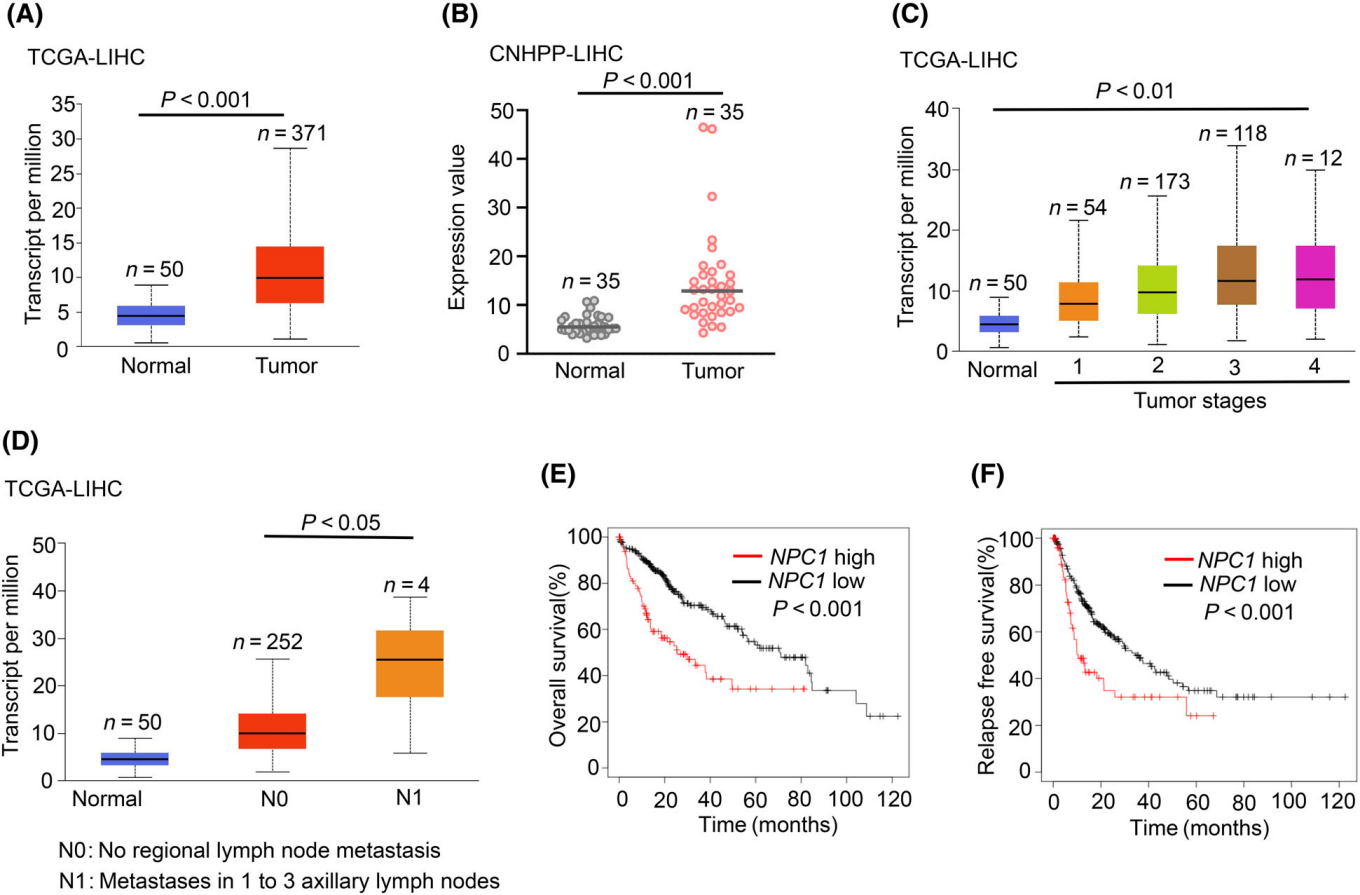
NPC1 mRNA is highly expressed in hepatocellular carcinoma and is associated with the progression of the disease and poor prognosis in patients. (A, B) The mRNA expression of NPC1 was examined by utilizing data from the TCGA database (A) and the Chinese Human Proteome Project (CNHPP) (B), data presented as mean ± SD. (C) The mRNA expression of NPC1 across different stages of hepatocellular carcinoma, again referencing the TCGA database, data presented as mean ± SD. (D) The mRNA expression of NPC1 in various metastasis stages of hepatocellular carcinoma, employing data from the TCGA database, data presented as mean ± SD. (E, F) The overall and relapse‐free survival rates of LIHC patients, categorizing them based on the expression levels of NPC1 as per the TCGA database records. For (A–D) statistical analyses were performed using an unpaired Student's *t*‐test, and (E, F) statistical analyses were performed using Log‐rank (Mantel–Cox) test, and the differences were considered statistically significant at *P* < 0.05.

To explore the relationship between NPC1 mRNA and the development of hepatocellular carcinoma, we initially analyzed the expression levels of NPC1 mRNA in normal liver tissues and hepatocellular carcinoma tissues. According to the data from the TCGA database, there was a significant upregulation of NPC1 mRNA in hepatocellular carcinoma tissues compared to normal tissues (Fig. 1A). Similarly, data analysis from the Chinese Human Proteome Project (CNHPP) also yielded comparable results (Fig. 1B). We then examined the expression levels of NPC1 mRNA during the progression of hepatocellular carcinoma and found that, as the cancer advanced, the expression levels of NPC1 mRNA gradually increased (Fig. 1C). In parallel, we observed an upward trend in the expression of NPC1 mRNA with the metastasis of hepatocellular carcinoma (Fig. 1D). The expression levels of NPC1 mRNA appeared to be unrelated to the patient's race, gender, age, and weight (Fig. S1B–E). Therefore, the ensuing critical question is whether the high expression of NPC1 mRNA is related to the prognosis of patients. By plotting survival curves correlating NPC1 mRNA expression levels with patient survival duration, we noticed that high expression of NPC1 mRNA is associated with overall poorer prognosis and shorter progression‐free periods for patients (Fig. 1E,F). In summary, our findings suggest that the high expression of NPC1 mRNA in hepatocellular carcinoma tissues holds significant implications.

### The protein levels of NPC1 are highly expressed in hepatocellular carcinoma and are related to poor prognosis in patients

**Fig. 2:**
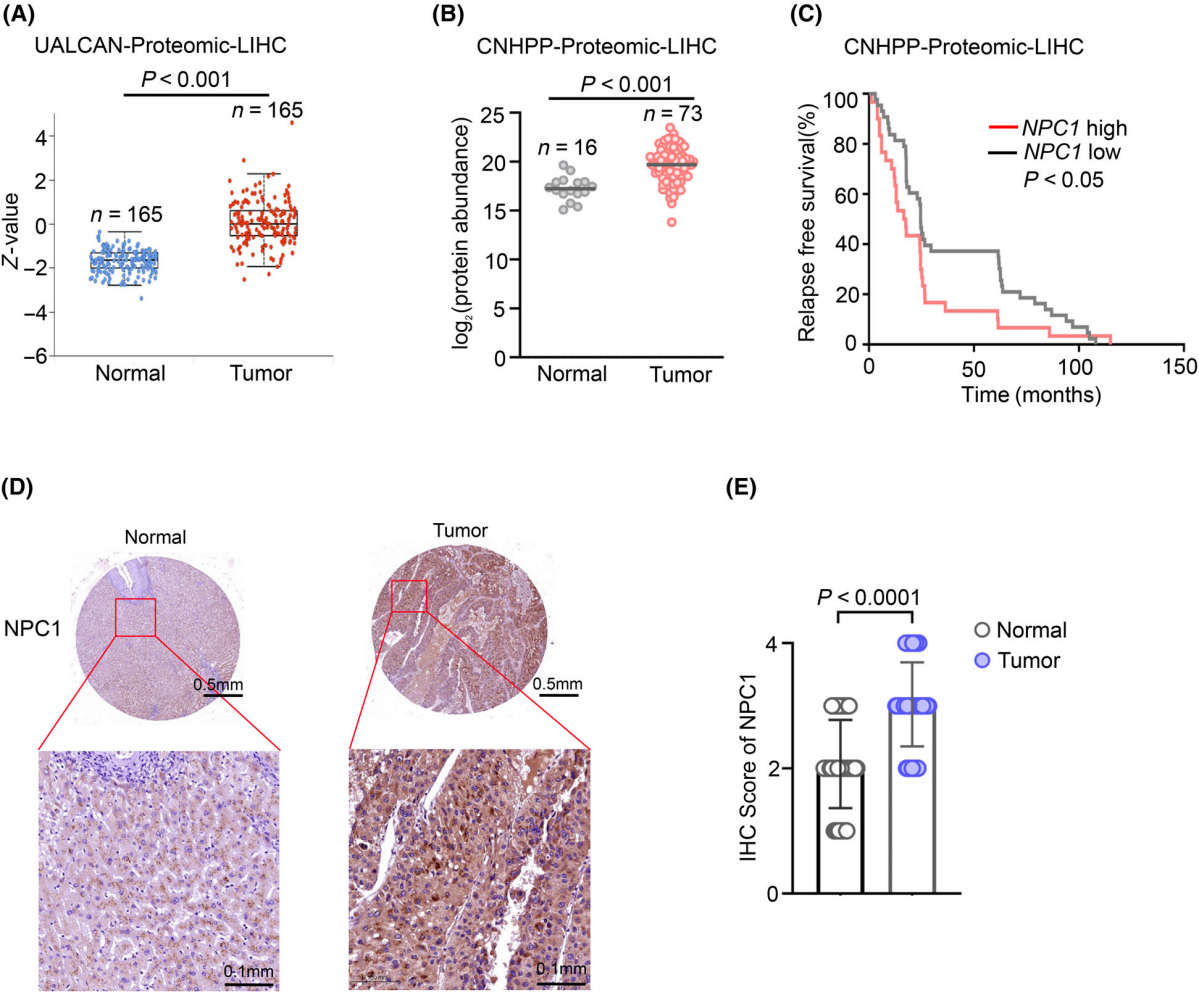
The protein levels of NPC1 are highly expressed in hepatocellular carcinoma and are related to poor prognosis in patients. (A, B) An analysis of the protein expression of NPC1 in LIHC was conducted utilizing resources from the TCGA database (using the online platform UALCAN and selecting Jitter Plot) and data from the Chinese Human Proteome Project (CNHPP), data presented as mean ± SD. (C) The relapse‐free survival curves of LIHC patients from the CNHPP database were stratified dependent on the expression of NPC1. (D) To further our understanding, we performed immunohistochemical staining on hepatocellular carcinoma and normal tissue samples was performed using an NPC1 antibody. (E) The IHC score of NPC1 in hepatocellular carcinoma cases was calculated, data presented as mean ± SD. For (A, B, E), statistical analyses were performed using an unpaired Student's *t*‐test, and (C) statistical analyses were performed using Log‐rank (Mantel–Cox) test, and the differences were considered statistically significant at *P* < 0.05.

To further explore the connection between NPC1 expression and hepatocellular carcinoma, we proceeded to evaluate the protein levels of NPC1 within hepatocellular carcinoma samples. Utilizing data from the UALCAN database on hepatocellular carcinoma, we observed a high expression of NPC1 protein in hepatocellular carcinoma tissues (Fig. 2A). Furthermore, employing data from the CNHPP database and dataset previously published [6], we obtained similar findings (Fig. 2B, Fig. S2A). Subsequently, we examined the relationship between the protein expression levels of NPC1 and patient prognosis. The results indicated that patients with high expression of NPC1 had poor prognoses (Fig. 2B). Moreover, through using the Proteinatlas database, we further confirmed the elevated expression of NPC1 in hepatocellular carcinoma tissues, reinforcing our earlier findings (Fig. S2B,C).

To validate the high expression of NPC1 in hepatocellular carcinoma tumor tissues, we collected clinical samples from 50 hepatocellular carcinoma patients and performed immunohistochemical staining for NPC1. By scoring the expression level of NPC1 (Fig. S2D), the results demonstrated high expression of NPC1 in the clinical samples of hepatocellular carcinoma patients as well (Fig. 2C,D). In summary, our results indicate that NPC1 protein is highly expressed in the tumor tissues of hepatocellular carcinoma.

### Knocking down NPC1 inhibits the proliferation of hepatocellular carcinoma cells *in vivo*

**Fig. 3:**
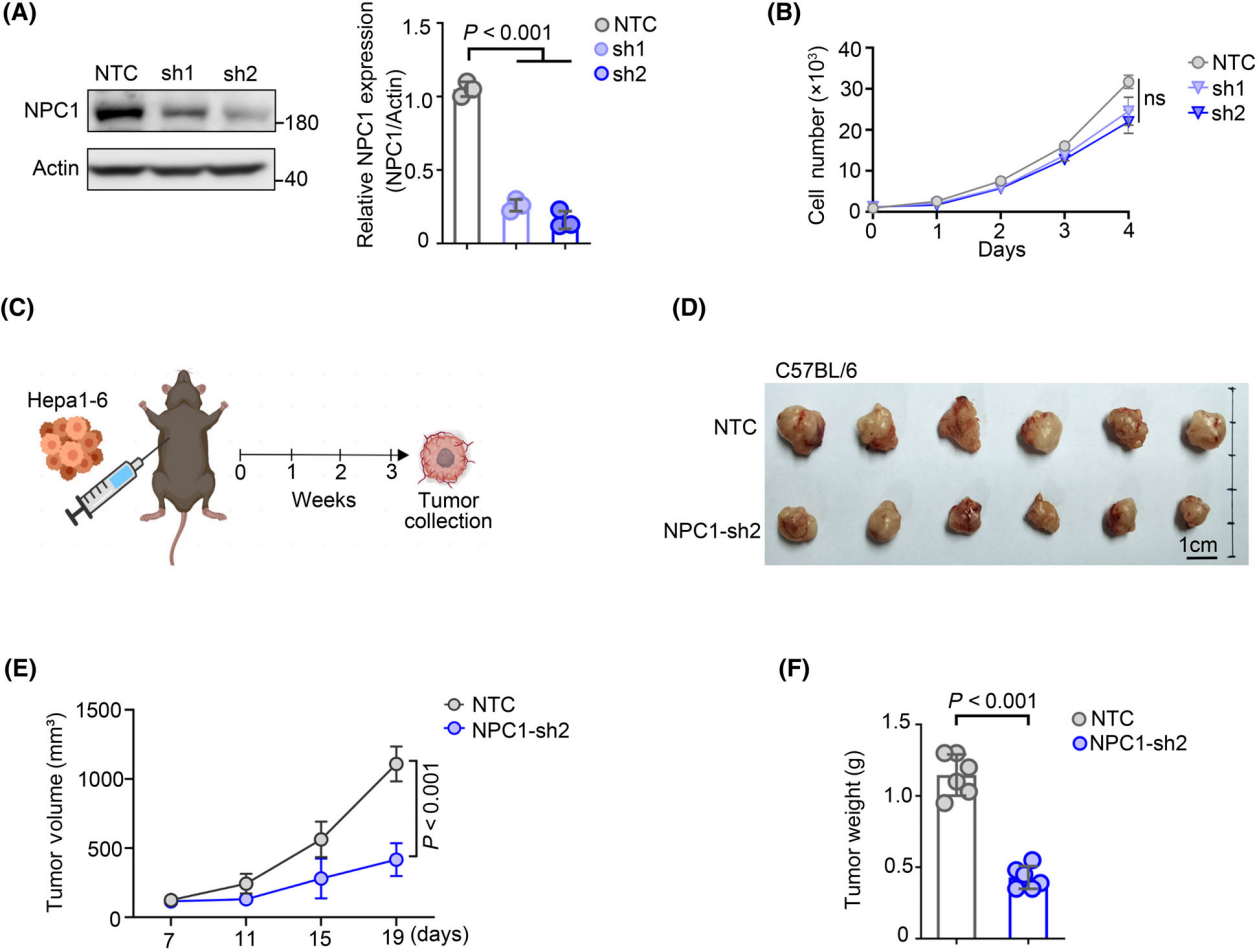
Knocking down NPC1 inhibits the proliferation of hepatocellular carcinoma cells *in vivo*. (A) Detection of NPC1 knockdown effects in Hepa1‐6 stable cell lines, followed by quantification of knockdown efficiency, data presented as mean ± SD. (B) Investigating the effects of NPC1 knockdown on the proliferation of Hepa1‐6 cells, data presented as mean ± SD. (C) Flowchart of the Hepa1‐6 mouse experiment. (D) Presentation of tumor images from the Hepa1‐6 mouse experiment. (E) Tumor growth curve diagram of the Hepa1‐6 mouse experiment, data presented as mean ± SEM. (F) Quantification of tumor weight outcomes from the Hepa1‐6 mouse experiment, data presented as mean ± SD. For (A, B, E, F), statistical analyses were performed using an unpaired Student's *t*‐test, and the differences were considered statistically significant at *P* < 0.05.

From our previous analysis and experiments, we observed a high expression of NPC1 in liver tumor. The subsequent issue is whether elevated levels of NPC1 participate in the control of liver tumor progression. In order to explore this question, we developed a Hepa1‐6 cell line with stable knockdown of NPC1 (Fig. 3A). Next, we examined whether knocking down NPC1 would affect intracellular cholesterol metabolism. Filipin staining showed that interfering with NPC1 leads to cholesterol accumulation within the cells, indicating that the knockdown of NPC1 is indeed sufficient to cause alterations in the cellular cholesterol metabolism pathway (Fig. S3A).

Subsequently we assessed NPC1's role in the proliferation of this liver tumor cell line (Fig. 3B), results indicated that knockdown of NPC1 by itself did not impact the *in vitro* proliferation of liver tumor cells (Fig. 3B). Considering the influence of the tumor microenvironment on hepatocellular carcinoma progression, we proceeded with *in vivo* mouse tumor transplantation experiments (Fig. 3C). The finding showed that knockdown of NPC1 significantly suppressed the *in vivo* progression of liver cells (Fig. 3D–F). Overall, our data suggest that elevated NPC1 expression facilitates the *in vivo* proliferation of liver tumor cells.

### NPC1 promotes hepatocellular carcinoma progression dependent on the infiltration of neutrophils

**Fig. 4:**
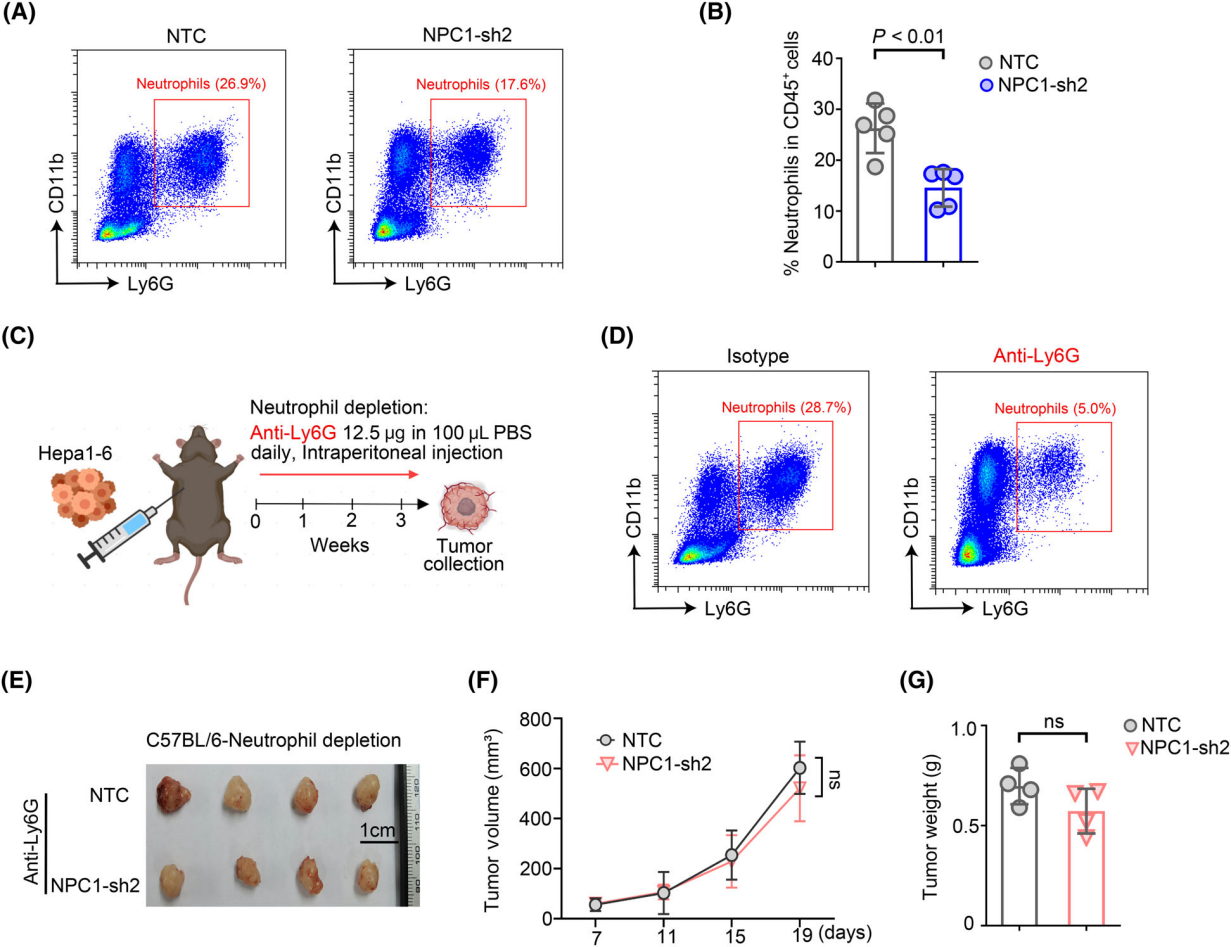
NPC1 promotes hepatocellular carcinoma progression dependent on the infiltration of neutrophils *in vivo*. (A) Flow cytometry measures the content of neutrophils in tumor tissues. (B) Quantitative results of (A), data presented as mean ± SD. (C) Flowchart of neutrophils depletion in the Hepa1‐6 mouse model. (D) Detection of neutrophils depletion effect in Hepa1‐6 mouse model using flow cytometry. (E) Presentation of tumor images from the Hepa1‐6 mouse experiment. (F) Tumor growth curve diagram of the Hepa1‐6 mouse experiment, data presented as mean ± SEM. (G) Quantification of tumor weight outcomes from the Hepa1‐6 mouse experiment, data presented as mean ± SD. For (B, F, G), statistical analyses were performed using an unpaired Student's *t*‐test, and the differences were considered statistically significant at *P* < 0.05.

As NPC1 itself does not significantly affect the growth of hepatocellular carcinoma tumor cells (Fig. 3A,B), implying that NPC1 in tumor cells might influence the progression of hepatocellular carcinoma via the tumor microenvironment. Cholesterol metabolism is positively correlated with tumor immune suppression. Notably, the previously work showed that NPC1 has a strong positive co‐expression with several immune‐suppressive genes in tumor tissues. Since macrophages and neutrophils are the main immunosuppressive cells in the tumor microenvironment, we examined their infiltration in tumor tissues after interfering with NPC1 in tumor cells. Our results revealed a significant reduction in neutrophils (Fig. 4A,B and Fig. S4A), while macrophages showed no significant difference (Fig. S4A,B), leading us to focus on NPC1's role in regulating neutrophil infiltration. Neutrophils are increasingly recognized as significant players in the development of liver tumor [23, 28]. An expanding evidence base positions neutrophils as crucial mediators of the immunosuppressive backdrop against which some cancers emerge, as well as catalysts for tumor advancement [28, 29]. This outcome suggests that NPC1 might advance the progression of hepatocellular carcinoma by encouraging the recruitment of neutrophils.

Subsequently, to explore whether the progression of hepatocellular carcinoma promoted by NPC1 is dependent on neutrophil recruitment, we conducted *in vivo* neutrophil depletion experiments (Fig. 4C). Flow cytometry results confirmed that neutrophils were effectively depleted *in vivo* with Anti‐Ly6G antibodies (Fig. 4D), after which we monitored tumor growth. Additionally, we examined the presence of anti‐tumor immune cells, specifically CD8^+^ T cells (Fig. S4C). In line with previous studies [30], neutrophil depletion in liver cancer tumors led to a significant increase in the number of CD8^+^ T cells within the tumor tissues (Fig. S4C). Our findings reveal that neutrophil depletion significantly curtailed hepatocellular carcinoma tumor progression, with tumor volumes and weights decreasing by approximately 50% (Fig. 4E–G) relative to scenarios where neutrophils were intact (Fig. 3D–F). Concurrently, following neutrophil depletion, downregulating NPC1 ceased to influence hepatocellular carcinoma progression (Fig. 4E–G). In summary, our findings confirm that the progression of hepatocellular carcinoma promoted by NPC1 relies on regulating neutrophil recruitment within the tumor microenvironment.

### High expression of NPC1 positively correlate with the extent of neutrophils infiltration in tumor tissues and poor prognosis of patients

**Fig. 5:**
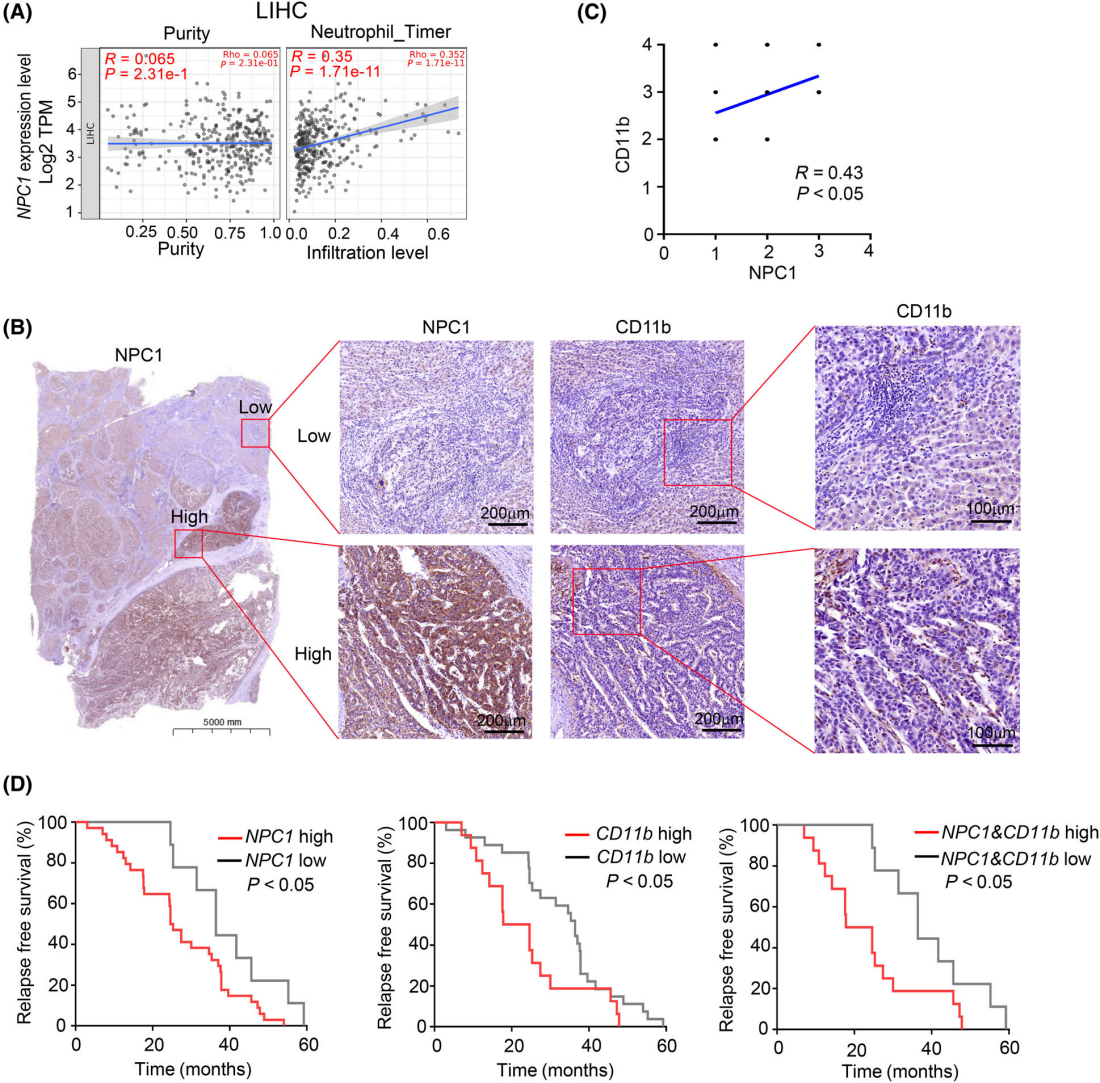
High expression of NPC1 positively correlate with the extent of neutrophil infiltration in tumor tissues and poor prognosis of patients. (A) Summary of the correlation between NPC1 expression with the infiltration level of neutrophil cells in LIHC. The relationship was estimated by TIMER2.0 using the TCGA database. (B) NPC1 and CD11b staining of the hepatocellular carcinoma tissue continuous sections. (C) Analysis of the correlation between NPC1 expression and the percentage of CD11b‐positive cells. (D) The survival curves illustrating the relationship between the expression levels of NPC1 and CD11b and the prognosis of hepatocellular carcinoma patients. For (C), statistical analyses were performed using the Pearson correlation coefficient, and (D) statistical analyses were performed using Log‐rank (Mantel–Cox) test, and the differences were considered statistically significant at *P* < 0.05.

In order to investigate the association between NPC1 and neutrophils in hepatocellular carcinoma tissues from clinical patients, we utilized the TCGA database for analysis (Fig. 5A and Fig. S4A). The results showed that the expression level of NPC1 was significantly positively correlated with the infiltration of neutrophils in hepatocellular carcinoma tissues (Fig. 5A and Fig. S4A). To further validate these results, we collected clinical samples from 50 hepatocellular carcinoma patients and performed consecutive sectioning and immunohistochemical staining for NPC1 and the neutrophil marker CD11b (Fig. 5B). The results showed that there was indeed a significant positive correlation between their expression levels in hepatocellular carcinoma patient tissues (Fig. 5B,C). Subsequently, we stratified patients based on the expression levels of NPC1 and CD11b and plotted the survival curves (Fig. 5D). The results indicated that, whether alone or in combination, high expression of NPC1 and CD11b was significantly positively correlated with poor survival in hepatocellular carcinoma patients (Fig. 5D). Overall, our findings indicate that the high expression of NPC1 could contribute to hepatocellular carcinoma progression by promoting neutrophil infiltration.

## Discussion

Hepatocellular carcinoma remains one of the major tumors threatening human health and life [2]. Despite various treatment methods available, such as surgery, chemotherapy, and radiotherapy, the effectiveness of these treatments for advanced hepatocellular carcinoma is limited. It is crucial to deeply analyze the mechanisms of hepatocellular carcinoma development, identify new biomarkers for early diagnosis, and discover novel treatment strategies or targets [31]. Our research found that the NPC1 protein, which is involved in cholesterol transport within cells, is highly expressed in hepatocellular carcinoma tissues and is associated with the progression and poor prognosis of hepatocellular carcinoma. The high expression of NPC1 ultimately promotes hepatocellular carcinoma progression by affecting the infiltration of neutrophils in the tumor microenvironment.

While interfering with NPC1 *in vitro* does not affect tumor cell growth, interference *in vivo* significantly inhibits tumor growth. This led us to hypothesize that NPC1 might regulate tumor progression through the tumor microenvironment. Based on literature, cholesterol metabolism is positively correlated with tumor immune suppression [32]. Notably, the data in the Nature article showed that NPC1 and several immune‐suppressive genes are highly expressed in tumor tissues [5]. Since macrophages and neutrophils are the main immunosuppressive cells in the tumor microenvironment [33, 34], we examined their infiltration in tumor tissues after interfering with NPC1 in tumor cells. Our results revealed a significant reduction in neutrophils, while macrophages showed no significant difference, leading us to focus on NPC1's role in regulating neutrophil infiltration. After depleting Ly6G, we primarily examined the number of anti‐tumor immune cells (CD8^+^ T cells). Consistent with previous studies, neutrophil depletion in liver cancer tumors significantly increased the number of CD8^+^ T cells in tumor tissues [35]. Neutrophils may promote liver cancer progression by regulating CD8^+^ T cell survival and recruitment [30].

We know that cholesterol is a crucial lipid molecule, essential for the structure and function of cell membranes, and also serves as a precursor for the synthesis of steroid hormones, bile acids, and vitamin D [36]. The liver is the central organ for cholesterol metabolism, responsible for its synthesis, storage, and secretion into the bloodstream [37]. Cholesterol metabolism is closely related to the progression of hepatocellular carcinoma, but early studies mainly focused on enzymes that directly regulate cholesterol metabolism, such as HMG‐CoA reductase [36]. The NPC1 gene, located on chromosome 18, plays a vital role in intracellular cholesterol transport [38]. Specifically, the NPC1 protein encoded by this gene is found in the membranes of lysosomes and endosomes, organelles responsible for digesting and recycling various substances [7, 9]. Although NPC1 is essential for cholesterol transport, its role in hepatocellular carcinoma progression is not well understood. Our preliminary research confirms a positive correlation between NPC1 and hepatocellular carcinoma progression.

Several cancer‐related mechanisms in relation to NPC1 could influence leucocyte recruitment [11]. The mechanisms include matrix metalloprotease (MMP) secretion, chaperone‐mediated autophagy or mTORC1 signaling [39], focal adhesion assembly [40, 41], Src localization and signaling, cholesterol‐sensitive SNARE functioning in exocytic pathways [42], caveolae formation [43], and fibronectin secretion [12]. Several of those aspects could influence leucocyte recruitment. Furthermore, NPC1 inhibition downregulates integrin cell surface expression [44], which is known to influence the adhesive properties of cells, include leucocytes. Additionally, cholesterol is considered indispensable for cell motility [40]. Previous studies have shown that the absence of NPC1 in tumor cells leads to cholesterol accumulation within the cells, reducing cholesterol levels in the tumor microenvironment [32, 45]. This, in turn, limits the cholesterol available to other cells in the microenvironment, impacting their functionality—particularly migration [41, 46]. Cholesterol is crucial for neutrophil polarization and chemotactic movement, and its depletion impairs neutrophils' ability to polarize and migrate toward chemoattractant [35]. We examined the correlation between NPC1 and the expression of molecules involved in neutrophil migration and recruitment including CXCR2 [47], Lsc [18], and myo1f [48]. Analysis of the TCGA database revealed that NPC1 expression is positively correlated with several genes linked to neutrophil migration (Fig. S5A). Therefore, it is evident that NPC1 may regulate neutrophil migration to tumor tissues through multiple pathways. Further experiments are needed to elucidate the mechanisms by which NPC1 influences neutrophil migration.

The progression of hepatocellular carcinoma is indeed accompanied by the remodeling of the immune microenvironment [4]. NPC1, a transmembrane protein located on lysosomal and late endosomal membranes, mainly functions in transporting cholesterol from lysosomes to the cytoplasmic membrane. Changes in the components of the cytoplasmic membrane inevitably affect intercellular signaling, thus influencing the tumor microenvironment. NPC1 may promote hepatocellular carcinoma progression by facilitating the infiltration of immunosuppressive cells in the tumor microenvironment. Neutrophils play a significant role in the development and progression of hepatocellular carcinoma [23, 28]. These cells promote tumor growth and metastasis through their immunosuppressive functions. Our findings demonstrate that NPC1 is capable of enhancing the infiltration of neutrophils within the hepatocellular carcinoma tumor microenvironment, and the infiltration of neutrophils is positively correlated with the poor prognosis of patients. Using further neutrophil depletion experiments, we determined that the role of NPC1 in advancing hepatocellular carcinoma progression truly relies on neutrophils. Therefore, the high expression of NPC1 may promote hepatocellular carcinoma progression by facilitating the infiltration of neutrophils in the tumor microenvironment.

## Conclusion

In this study, we systematically explored the relationship between NPC1 and hepatocellular carcinoma. Using various sources of hepatocellular carcinoma data and immunohistochemical staining of tumor tissues, we confirmed that NPC1 is highly expressed in hepatocellular carcinoma tissues and is associated with poor prognosis in patients. Preliminary mechanistic exploration suggests that NPC1 promotes hepatocellular carcinoma progression by facilitating the infiltration of neutrophils in the tumor microenvironment. Our research has provided new potential targets for the diagnosis and treatment of hepatocellular carcinoma.

## Conflict of interest

The authors declare no conflict of interest.

### Peer review

The peer review history for this article is available at https://www.webofscience.com/api/gateway/wos/peer‐review/10.1002/2211‐5463.13951.

## Author contributions

FL and SY conceived and designed the project, SY, JC, and KX acquired the data. FL and SY analyzed and interpreted the data, SY and FL wrote the paper.

## Supporting information


**Fig. S1.** NPC1 mRNA is highly expressed in hepatocellular carcinoma and is associated with the progression of the disease and poor prognosis in patients.

**Fig. S2.** The protein levels of NPC1 are highly expressed in hepatocellular carcinoma and are related to poor prognosis in patients.

**Fig. S3.** Cholesterol accumulation in Hepa1‐6 cells transfected with NPC1‐sh1 and NPC1‐sh2 was detected using filipin staining, and the intensity was quantified.

**Fig. S4.** The gating strategy and quantification data of immune cells in tumor microenvironments.

**Fig. S5.** Summary of the correlation between NPC1 expression with the expression of CXCR2, Lsc and Myo1f using the TCGA date in the Ualcan website.

## Data Availability

The data that support the findings of this study are available from the corresponding author [liufubao@ahmu.edu.cn] upon reasonable request.
