# Supplementary material for: NPC1 promotes the progression of hepatocellular carcinoma by mediating the accumulation of neutrophils into the tumor microenvironment
Source: FEBS Open Bio. 2024 Dec 20;15(4):661–73. doi: 10.1002/2211-5463.13951 (PMC11961396; doi:10.1002/2211-5463.13951)
Supplement: Supplementary file 1 — Fig. S1. NPC1 mRNA is highly expressed in hepatocellular carcinoma and is associated with the progression of the disease and poor prognosis in patients. [file FEB4-15-661-s004.docx]

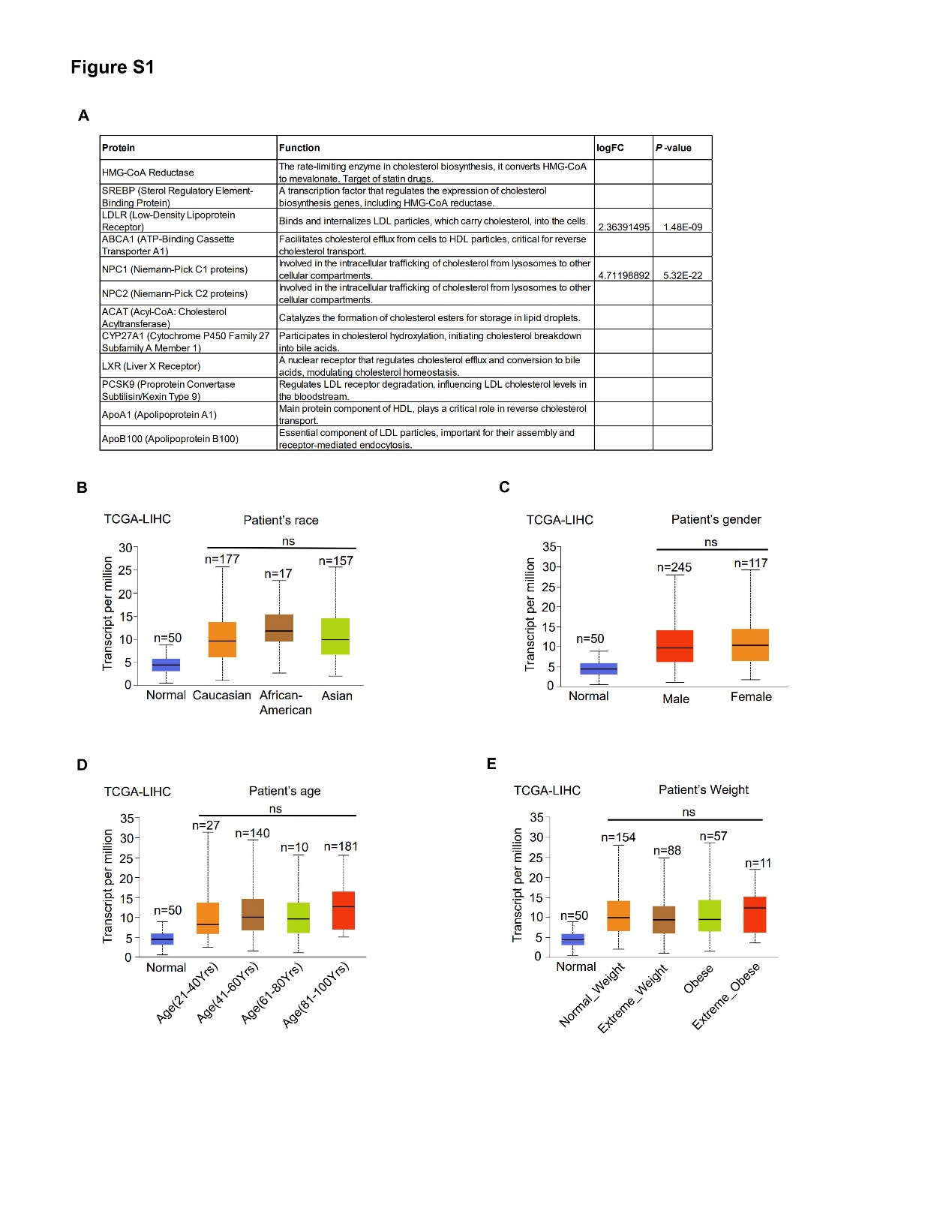


Figure S1. NPC1 mRNA is highly expressed in hepatocellular carcinoma and is associated with the progression of the disease and poor prognosis in patients. (A) The key proteins involved in cholesterol metabolism pathways and their expression levels in tumor tissues compared to normal tissues. LogFC represents the log2 ratio of gene expression in tumor tissue compared to normal tissue. (B, C, D, E) The mRNA expression of NPC1 in different patient’s race, gender, age and weight of hepatocellular carcinoma was analyzed using the TCGA database. All data presented as mean ± SD. For (B-E) statistical analyses were performed using an unpaired Student’s t-test, and the differences were considered statistically significant at P < 0.05.
