# Supplementary material for: NPC1 promotes the progression of hepatocellular carcinoma by mediating the accumulation of neutrophils into the tumor microenvironment
Source: FEBS Open Bio. 2024 Dec 20;15(4):661–73. doi: 10.1002/2211-5463.13951 (PMC11961396; doi:10.1002/2211-5463.13951)
Supplement: Supplementary file 2 — Fig. S2. The protein levels of NPC1 are highly expressed in hepatocellular carcinoma and are related to poor prognosis in patients. [file FEB4-15-661-s002.docx]

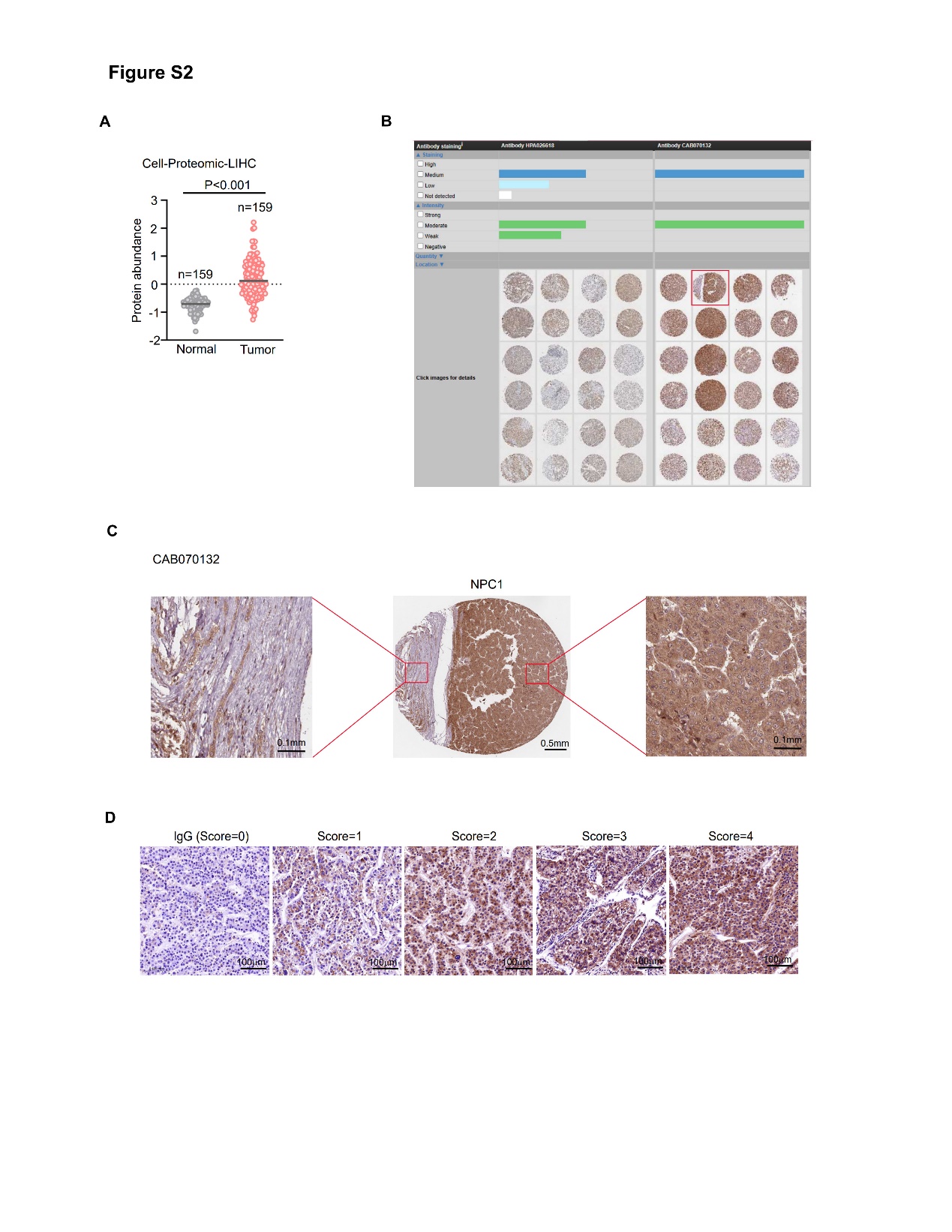


Figure S2. The protein levels of NPC1 are highly expressed in hepatocellular carcinoma and are related to poor prognosis in patients. (A) The protein expression of NPC1 in LIHC was analyzed using the data in the cell paper, data presented as mean ± SD. (B, C) Immunohistochemical staining of Hepatocellular carcinoma and normal tissue using NPC1 antibody, this data from ProteinAltas. (D) The standard of IHC score of NPC1 in hepatocellular carcinoma. For (A) statistical analyses were performed using an unpaired Student’s t-test, and the differences were considered statistically significant at P < 0.05.
