# Supplementary material for: NPC1 promotes the progression of hepatocellular carcinoma by mediating the accumulation of neutrophils into the tumor microenvironment
Source: FEBS Open Bio. 2024 Dec 20;15(4):661–73. doi: 10.1002/2211-5463.13951 (PMC11961396; doi:10.1002/2211-5463.13951)
Supplement: Supplementary file 3 — Fig. S3. Cholesterol accumulation in Hepa1‐6 cells transfected with NPC1‐sh1 and NPC1‐sh2 was detected using filipin staining, and the intensity was quantified. [file FEB4-15-661-s001.docx]

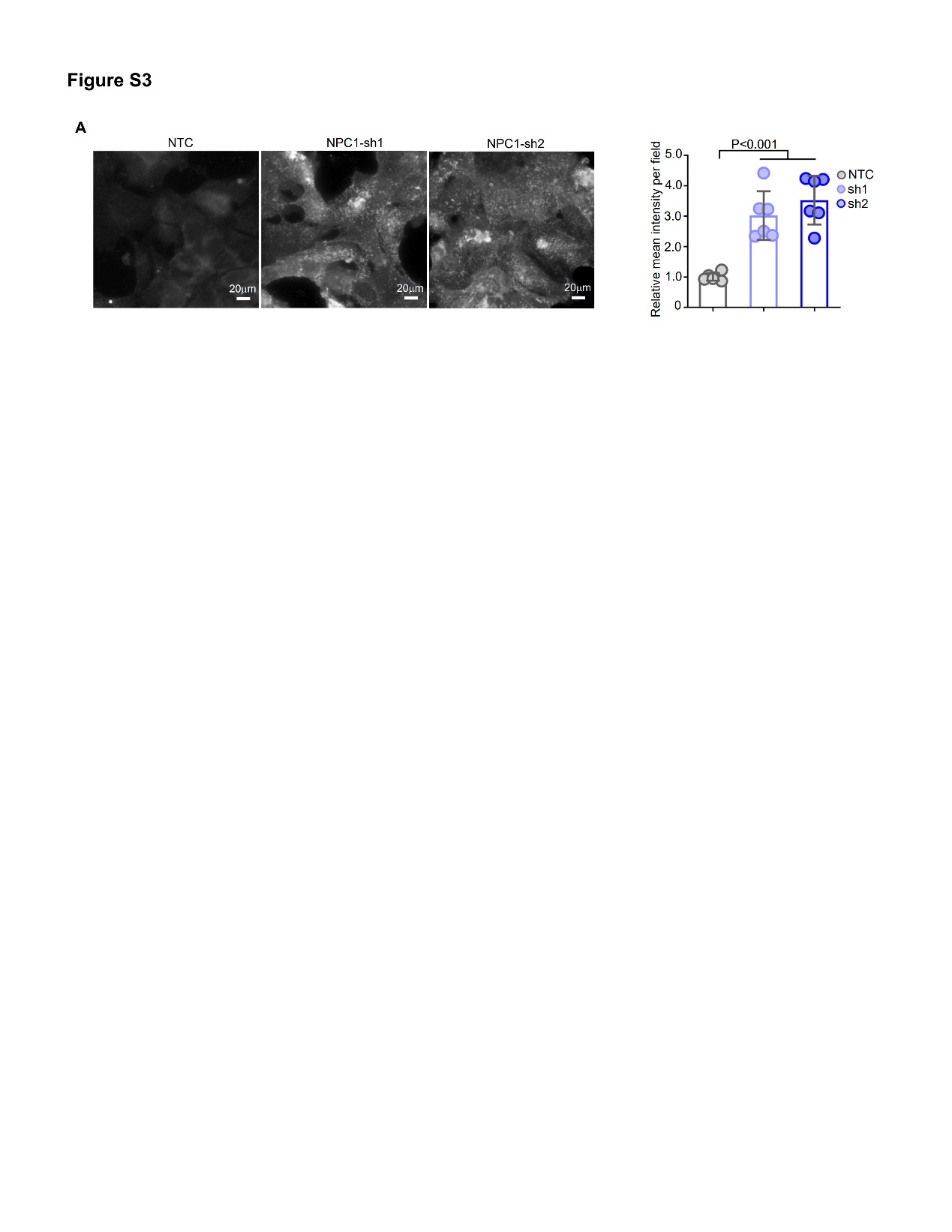


Figure S3. Cholesterol accumulation in Hepa1-6 cells transfected with NPC1-sh1 and NPC1-sh2 was detected using filipin staining, and the intensity was quantified, data presented as mean ± SD. Three independent experiments were performed, for each experiment the intensities of at less six cells were calculated. Statistical analyses were performed using an unpaired Student’s t-test, and the differences were considered statistically significant at P < 0.05.
