# Supplementary material for: NPC1 promotes the progression of hepatocellular carcinoma by mediating the accumulation of neutrophils into the tumor microenvironment
Source: FEBS Open Bio. 2024 Dec 20;15(4):661–73. doi: 10.1002/2211-5463.13951 (PMC11961396; doi:10.1002/2211-5463.13951)
Supplement: Supplementary file 4 — Fig. S4. The gating strategy and quantification data of immune cells in tumor microenvironments. [file FEB4-15-661-s003.docx]

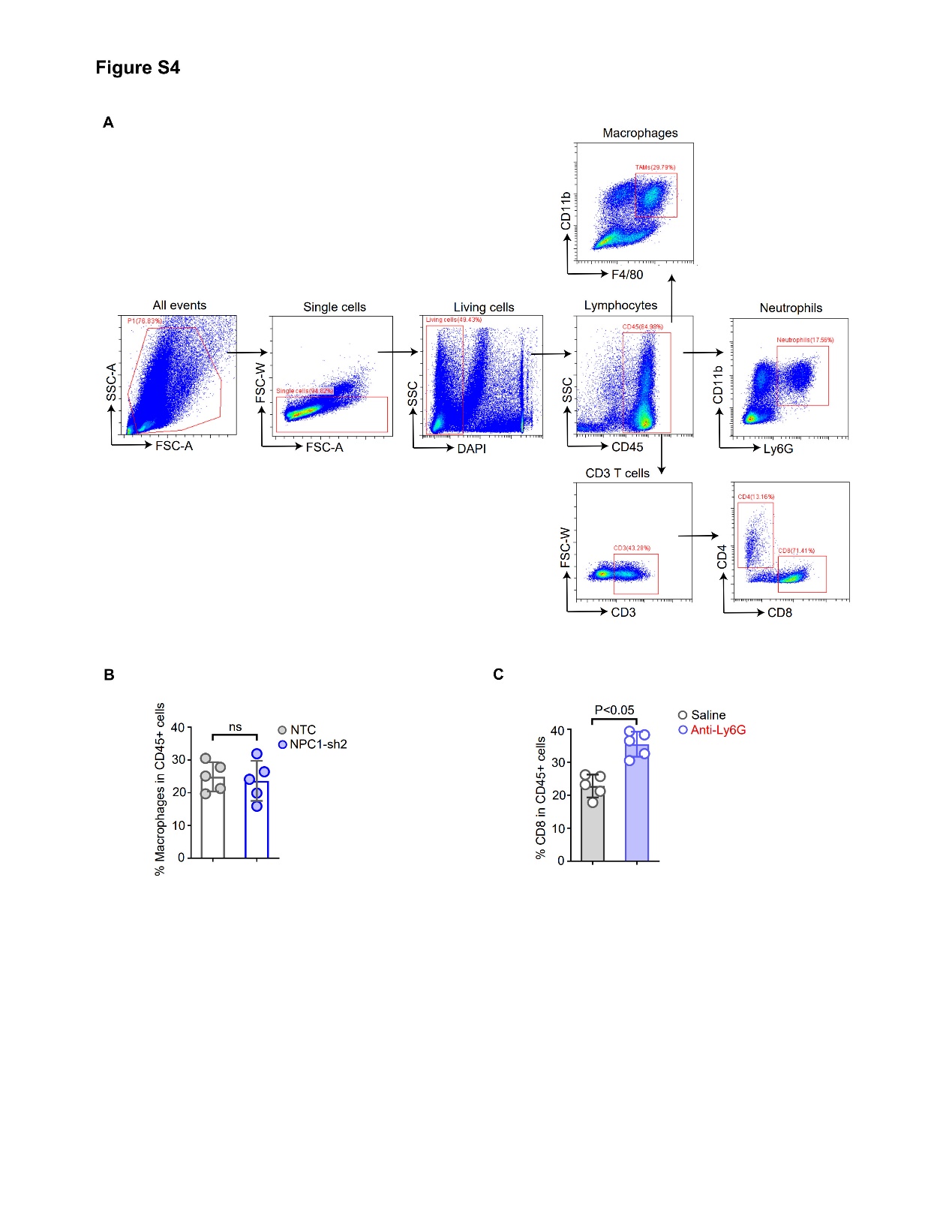


Figure S4. The gating strategy and quantification data of immune cells in tumor microenvironments. (A)The gating strategy for neutrophils, macrophages and CD8+ T cells in flow cytometry. (B) Quantification of macrophages in the Hepa1-6 mouse tumor, n=5, data presented as mean ± SD. (C) Quantification of CD8+ cells in the Hepa1-6 mouse tumor treated with saline and anti-Ly6G, n=5, data presented as mean ± SD. For (B-C) statistical analyses were performed using an unpaired Student’s t-test, and the differences were considered statistically significant at P < 0.05.
