# Supplementary material for: NPC1 promotes the progression of hepatocellular carcinoma by mediating the accumulation of neutrophils into the tumor microenvironment
Source: FEBS Open Bio. 2024 Dec 20;15(4):661–73. doi: 10.1002/2211-5463.13951 (PMC11961396; doi:10.1002/2211-5463.13951)
Supplement: Supplementary file 5 — Fig. S5. Summary of the correlation between NPC1 expression with the expression of CXCR2, Lsc and Myo1f using the TCGA date in the Ualcan website. [file FEB4-15-661-s005.docx]

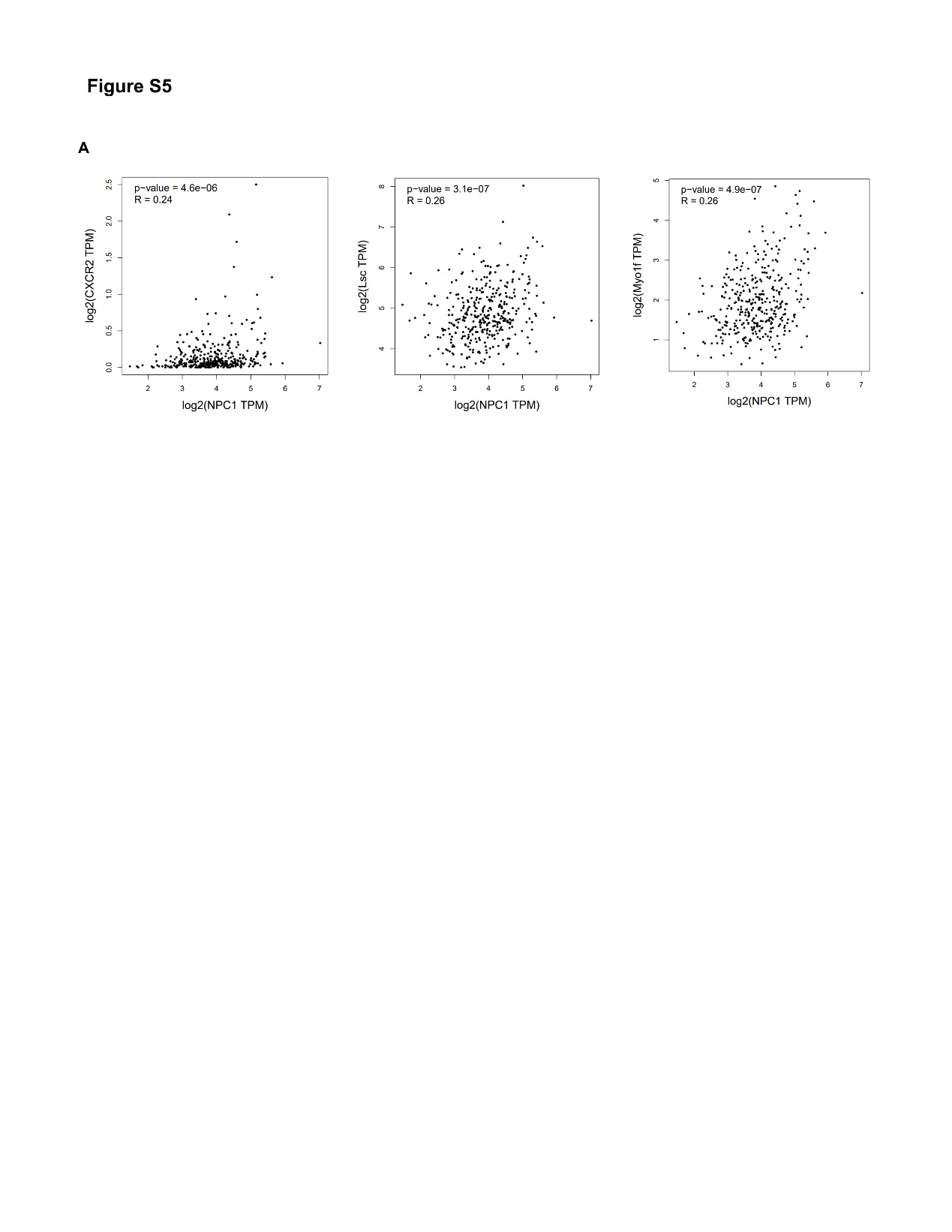


Figure S5. Summary of the correlation between NPC1 expression with the expression of CXCR2, Lsc and Myo1f using the TCGA date in the Ualcan website. Statistical analyses were performed using the Pearson correlation coefficient, and the differences were considered statistically significant at P < 0.05.
